# Editorial: Immune Regulation in Kidney Diseases: Importance, Mechanism and Translation, Volume II

**DOI:** 10.3389/fmed.2022.921987

**Published:** 2022-05-31

**Authors:** Cheng Yang, Bin Yang

**Affiliations:** ^1^Department of Urology, Zhongshan Hospital, Fudan University, Shanghai, China; ^2^Shanghai Key Laboratory of Organ Transplantation, Shanghai, China; ^3^Zhangjiang Institute of Fudan University, Shanghai, China; ^4^Department of Cardiovascular Sciences, College of Life Sciences, University of Leicester, University Hospitals of Leicester National Health Service (NHS) Trust, Leicester, United Kingdom; ^5^Department of Nephrology, Nantong-Leicester Joint Institute of Kidney Science, Affiliated Hospital of Nantong University, Nantong, China

**Keywords:** kidney, Immune Regulation, mechanism, acute kidney injury, kidney transplantation

The immune system is vital for survival in humans. It is responsible for the body's defense against infectious organisms, external pathogens and other invaders, while the immune response is regulated precisely, to prevent extensive damage to the body. However, this system sometimes malfunctions, misinterpreting the body's own tissues as foreign and produce antibodies or active immune cells to target and attack particular cells or tissues, resulting in autoimmune disease.

The kidneys contribute to immune homeostasis, while several key components of the immune response have been implicated in the progression of renal diseases. Although several mechanisms of immune dysregulation leading to renal disease is broad, the pathways leading to injury are similar. Loss of immune homeostasis in renal disease results in perpetual immune cell recruitment and worsening damage to the kidney. At the same time, uncoordinated attempts at tissue repair, after immune-mediated disease or non-immune mediated injury, result in fibrosis of structures important for renal function, leading eventually to kidney failure.

We were able to collect Volume II of *Immune Regulation in Kidney Diseases: Importance, Mechanism and Translation* (https://www.frontiersin.org/research-topics/10976/immune-regulation-in-kidney-diseases-importance-mechanism-and-translation). There are 12 papers in this issue, with 11 original research and one case report. Renal acute and chronic injury, kidney transplantation, infection, and immunological modulation are all included in this field.

In kidney transplantation, ischemia reperfusion (IR) injury is a preventable process and is linked to delayed graft function, rejection, and long-term allograft survival. In terms of innate immunity activation, inflammation and stress play a significant role in IR injury. Positive immunological responses, on the other hand, might lead to negative immune system activation. Mesenchymal stem cells (MSCs) have a strong and distinct immunosuppressive effect. They can be split into two types: pro-inflammatory phenotype and anti-inflammatory phenotype (also known as MSC I and MSC II). Chen et al. used poly (I:C) to produce anti-inflammatory MSCs that had better benefits in maintaining renal function, reducing tissue damage, and reducing systemic inflammation in IR. They discovered that anti-inflammatory MSCs had the powerful anti-inflammatory properties mentioned above injury. In previous study, Yan et al. discovered that SHP-1 deficiency exacerbates renal IR injury in a prior investigation (1). They used RNA-seq to compare the expression profiles of SHP-1 (encoded by Ptpn6)-deficient animals with wild-type mice in the work published in this issue. SHP-1 deficiency decreased the production of genes in the PPAR signaling pathway, resulting in increased ROS and aggravating renal IR injury, according to their findings (Yan et al.).

Two important pathophysiological processes in kidney IR injury are inflammation and cell death. Calpains, a Ca2+-dependent cysteine protease family, are important in the pathophysiology of renal disorders. Calpeptin, a calpain inhibitor, reduced AIM2 inflammasome activation and raised Klotho protein expression, protecting renal function in an IR damage model, according to Wu et al. In obese individuals, lipotoxicity in the kidneys contribute to renal cell injury and death. In obesity-related kidney disease, local renin-angiotensin system (RAS) activation may play a role. The cargo miR- 6869-5p in plasma extracellular vehicles (EVs) produced RAS activation and renal tubular injury, according to Liu et al. As a result, plasma miR-6869-5p in obesity-related kidney illness, obese-EVs could be a therapeutic target for local RAS activation. It is critical to select an appropriate experimental model for renal IR injury. Human pluripotent stem cells (hPSCs)-derived kidney organoids operate as a model of different types of kidney cells *in vitro* and eliminate potential confounders *in vivo*. Zhang et al. used human kidney organoids to create LPS-induced kidney damage models. These findings laid a solid foundation for kidney damage research.

Immunosuppression therapy is unavoidable in kidney transplant recipients. Because all immunosuppressive medicines are not specific to allografts, the immune system is typically inhibited. As a result, infection following transplantation is a common but difficult problem. In kidney transplant recipients, opportunistic infections like the BK virus are common. Wang et al. used multivariate logistic regression analysis and external validation to retrospectively examine clinical and laboratory variables associated with a higher risk of BK virus activation from 195 renal transplant recipients. In kidney transplant recipients, the innovative predictive nomogram accurately predicted BK virus activation. Severe pneumonia is a life-threatening complication that occurs after a kidney transplant and results in long-term immunosuppression. However, only a few immune-related markers are employed to determine the severity of pneumonia. Myeloid-derived suppressor cells (MDSCs) are an important immune regulatory cell that is induced during infection and has a significant immunosuppressive capacity. The relationship between MDSCs and pneumonia in kidney transplant recipients was investigated by Peng et al. They discovered that G-MDSCs were linked to the severity of pneumonia and may thus be used to measure pneumonia severity as an indicator of immunological function. Despite tremendous improvements in immunosuppression, the long-term result of juvenile kidney transplantation remains unsatisfactory. The 10-year graft survival rate is only about 60%. Donor-derived cell-free DNA (ddcfDNA) has emerged as a possible biomarker for graft damage and rejection detection. The dynamic features of ddcfDNA after juvenile kidney transplantation were revealed by Nie et al. We included an omics study on acute rejection in this issue. Using RNA sequencing, Wang et al. identified the peripheral blood mononuclear cells (PBMCs) of patients with acute renal allograft rejection. This issue also includes a case report in addition to the original clinical and fundamental studies mentioned above. Zhao et al. were the first to report a successful ABO-incompatible deceased donor kidney transplantation (ABOi DDKT) in a newborn. No pre- or post-transplantation antibody removal treatment was performed, since the recipient's anti-iso-hemagglutinin-A Ig- M/G antibody titers were both low (1:2) before transplantation and have remained at low levels or undetectable to date (Zhao et al.).

In diabetic patients, the levels of circulating tumor necrosis factor receptor (TNFR) 1 and 2 can predict decline in estimated glomerular filtration rate (eGFR). Gohda et al. examined the association between baseline TNFR levels, early febuxostat-induced TNFR changes, and future eGFR reduction in chronic kidney disease (CKD) patients without diabetes. C4d is a diagnostic marker for antibody-mediated rejection in kidney transplantation; however, Yang et al. found that the amount and pattern of renal C4d distribution differed between lupus nephritis (LN) and IgA nephropathy (IgAN) patients. They concluded that the presence of C4d in renal tissue acted as an independent predictor of relapse for LN patients and disease progression for IgAN patients (Yang et al.).

Finally, all of the manuscripts discussed immune modulation in acute, chronic, and kidney transplantation. We believe that this study area will lead to new insights into immune modulation in renal disease. All authors, reviewers, and editors who contributed are greatly appreciated. We believe readers enjoy this special issue.

## Author Contributions

CY and BY wrote the editorial. Both authors approved the submitted version.

## Conflict of Interest

The authors declare that the research was conducted in the absence of any commercial or financial relationships that could be construed as a potential conflict of interest.

## Publisher's Note

All claims expressed in this article are solely those of the authors and do not necessarily represent those of their affiliated organizations, or those of the publisher, the editors and the reviewers. Any product that may be evaluated in this article, or claim that may be made by its manufacturer, is not guaranteed or endorsed by the publisher.
